# Comparing cognitive behavioral therapy and social prescribing in patients with loneliness on long-term opioid therapy to reduce opioid misuse: protocol for a randomized controlled trial

**DOI:** 10.1186/s13722-024-00498-y

**Published:** 2024-09-11

**Authors:** Sebastian T. Tong, Kris Pui Kwan Ma, Ajla Pleho, Brennan Keiser, Chialing Hsu, Dawn M. Ehde, Mary C. Curran, Judith I. Tsui, Patrick J. Raue, Kari A. Stephens

**Affiliations:** 1 Department of Family Medicine, University of Washington, 4311 11th Ave NE, Suite 210, Seattle, WA 98105 USA; 2 Department of Rehabilitation Medicine, University of Washington, Seattle, USA; 3 Department of Psychiatry and Behavioral Sciences, University of Washington, Seattle, USA; 4 Department of Medicine, University of Washington, Seattle, USA

**Keywords:** Loneliness, Opioid misuse, Opioid-related disorders, Opioid analgesics, Psychosocial interventions

## Abstract

**Background:**

Patients with chronic pain on opioids frequently experience loneliness, which is associated with poorer health outcomes and higher risk for opioid misuse and opioid use disorder. Given that almost half of opioids are prescribed in primary care, a critical need exists for the development and testing of interventions to reduce loneliness in primary care patients at risk for opioid misuse. Cognitive behavioral therapy and social prescribing have been shown to be efficacious in reducing loneliness and improving outcomes in other populations but have not been tested in patients at risk for substance use disorder. The overall objective of our study is to reduce opioid misuse and opioid use disorder by addressing loneliness in patients on long-term opioid therapy in real-world primary care settings.

**Methods:**

We will conduct a 3-arm pragmatic, randomized controlled trial to compare the effectiveness of two group-based, telehealth-delivered interventions with treatment as usual: (1) cognitive behavioral therapy to address maladaptive thought patterns and behaviors around social connection and (2) a social prescribing intervention to connect participants with social opportunities and develop supportive social networks. Our primary outcome is loneliness as measured by the UCLA Loneliness Scale and our dependent secondary outcome is opioid misuse as measured by the Common Opioid Misuse Measure. We will recruit 102 patients on long-term opioid therapy who screen positive for loneliness from 2 health care systems in Washington State. Implementation outcomes will be assessed using the RE-AIM framework.

**Discussion:**

Our study is innovative because we are targeting loneliness, an under-addressed but critical social risk factor that may prevent opioid misuse and use disorder in the setting where most patients are receiving their opioid prescriptions for chronic pain. If successful, the project will have a positive impact in reducing loneliness, reducing opioid misuse, improving function and preventing substance use disorder.

**Trial Registration:**

NCT06285032, issue date: February 28, 2024, original.

## Background

Individuals on long-term opioid therapy frequently experience loneliness, which is associated with poorer health outcomes and higher risks for opioid misuse and opioid use disorder [1]. Loneliness, which refers to the perceived lack of connections to others and the feeling of not belonging [2] has increased substantially during the COVID-19 pandemic to greater than half of U.S. adults [3–6]. Loneliness as a mediating factor leading to substance use disorder is becoming increasingly relevant as rates of drug overdose, substance use disorder and opioid misuse continue to rise substantially, exacerbated by the COVID-19 pandemic and the resultant social and physical isolation [7–11]. Even before the pandemic, a brief decline in drug overdose deaths had reversed despite substantial efforts to reduce opioid prescribing in the United States [12]. Meanwhile, initial decreases in opioid prescribing have plateaued during the COVID-19 pandemic as barriers to non-pharmacologic modalities of treating chronic pain increased [13, 14] leading to worsening care for patients with chronic pain [15]. Many experts have hypothesized that the social distancing requirements initially put in place during the pandemic resulted in decreased social connection and increased loneliness resulting in increased risk for opioid misuse and overdose [16].

A 2023 U.S. Surgeon General Advisory highlighted the association of loneliness with poor health outcomes and proposed a national strategy for addressing loneliness [17]. This strategy includes 6 pillars, of which one concerns mobilization of the health care sector. This is relevant because individuals experiencing loneliness have greater healthcare utilization in both outpatient [18, 19] and acute care [20, 21] settings.

Primary care could play an important frontline role in reducing loneliness in those on long-term opioid therapy to potentially reduce risk for opioid misuse and opioid use disorder. As the most common and often first setting where individuals seek care [22], primary care treats over half of the patients with chronic pain and prescribes almost 50% of opioids nationwide [23, 24]. Our preliminary work [25, 26] and other studies [27] have suggested that at least 20% of primary care patients reported loneliness that impact their chronic conditions and functioning. As such, a primary care based intervention will have the highest likelihood of reaching the most patients to address loneliness and prevent opioid misuse and overdose. In addition, primary care focuses on prevention [28] and growing literature suggests that prevention of addiction (and the recognition of preaddiction as an important precursor to substance use disorders) is important and undervalued [29, 30].

The overall objective of our study is to adapt and test two evidence-based loneliness interventions, cognitive behavioral therapy (CBT) and social prescribing, with the goal of reducing opioid misuse and opioid use disorder in primary care patients with chronic pain. These two interventions have shown efficacy in reducing loneliness and improving health outcomes primarily in older adults [31, 32] but have yet to be tested for patients on long-term opioid therapy.

Our study’s specific aims are:

1. To determine the effectiveness of two group based, telehealth-delivered loneliness interventions (CBT and social prescribing) relative to usual care in reducing loneliness (primary outcome) and opioid misuse (secondary outcome) in a 3-arm randomized controlled trial.

### Hypothesis

We hypothesize that both CBT and social prescribing will be superior to usual care in reducing loneliness and that reductions in loneliness will then lead to reductions in opioid misuse. We hypothesize that CBT and social prescribing will be noninferior.

2) To assess implementation outcomes using the RE-AIM framework from a 3-arm randomized controlled trial testing the CBT and social prescribing interventions in primary care practices.

## Methods/design

### Study overview

**Fig. 1:**
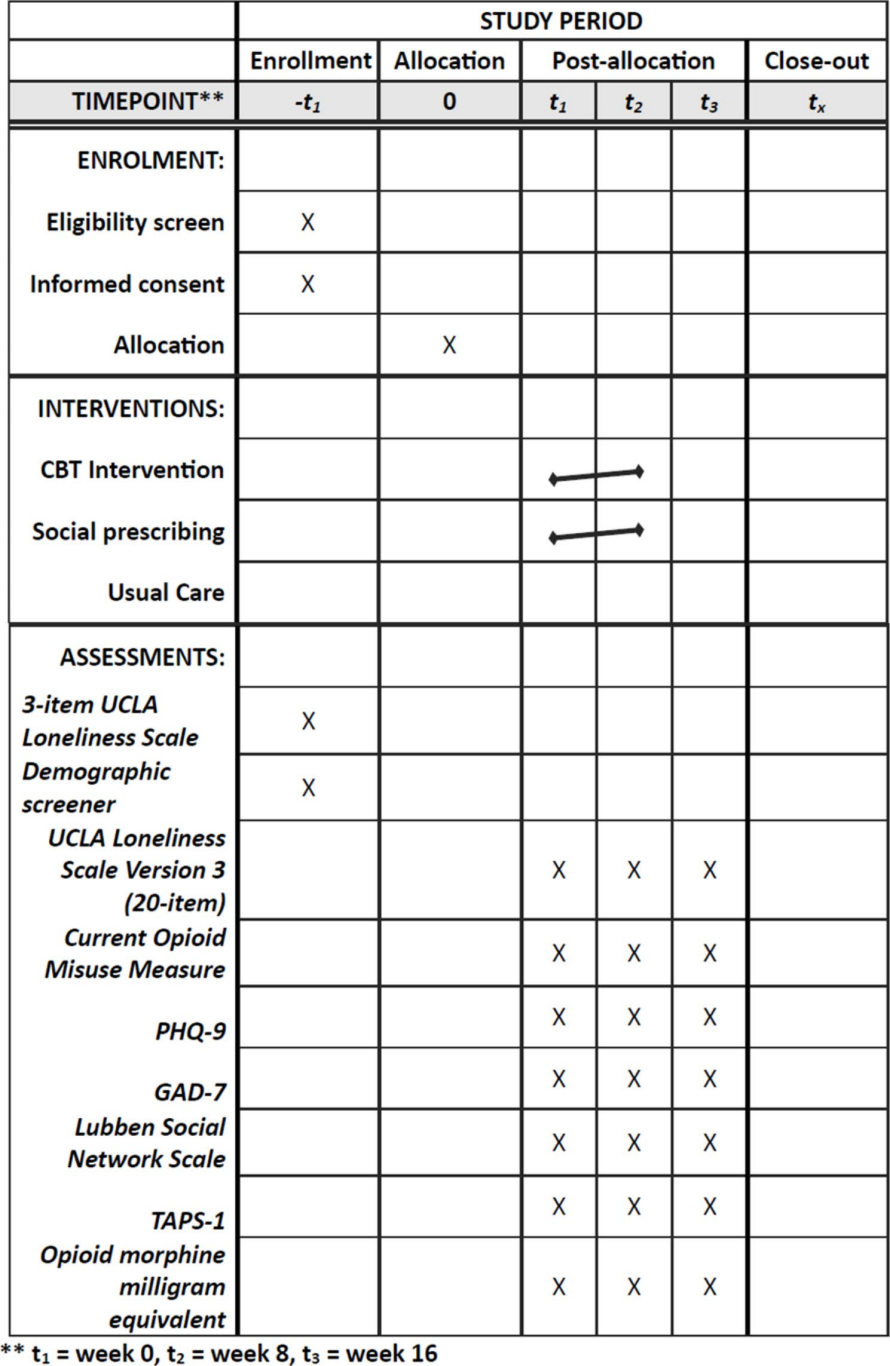
Schedule of enrollment, interventions, and assessments

We will conduct a 3-arm randomized controlled trial comparing two-group based, telehealth interventions for loneliness. Participants with loneliness on long-term opioid therapy will be randomized to one of the following arms: (1) CBT intervention, (2) social prescribing intervention, or (3) usual care. We will collect outcomes on loneliness (primary outcome), current opioid misuse, substance use, social connection, depression, anxiety, and function (secondary outcomes) post-intervention and at 3 months follow-up post-intervention. Figure 1 shows the schedule of enrolment, interventions, and assessments.

### Study framework

**Fig. 2:**
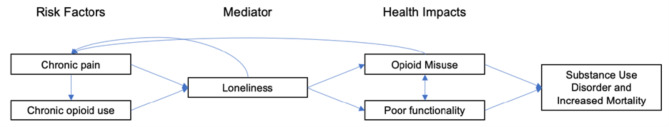
Study framework for loneliness and opioid misuse

Our study framework adapts the National Academies of Science, Engineering and Medicine framework on loneliness and social isolation [33] to understand the relationship between long-term opioid use/chronic pain and misuse/substance use disorder with loneliness as a mediating factor (Fig. 2). Specifically, we propose both chronic pain and long-term opioid use as related but unique risk factors for loneliness that can lead to opioid misuse and poor function followed by substance use disorder and overdose mortality risk. Our framework is supported by multiple studies that report on this cyclical association of chronic pain, long-term opioid use and loneliness [34–37].

### Participant eligibility

We will recruit patients who are on long-term opioid therapy (defined as ≥ 3 months receiving prescribed opioids [functional definition is ≥ 3 opioid prescriptions each ≥ 21 days apart]) and who meet the following inclusion criteria: English-speaking, 18 years of age or older and score 6 or greater on the 3-item UCLA-loneliness scale (the scale is scored from 3 to 9). The 3-item scale will be used for eligibility while the 20-item scale will be used for outcomes. We will exclude patients who have active cancer, active psychosis, or moderate/severe cognitive impairment using the mini-MOCA, are on palliative care or live in a controlled setting (i.e., assisted living, nursing home or inpatient treatment facility).

### Setting and participant recruitment

We plan to recruit from primary care clinics from 2 health systems that are part of our practice-based research network, the WWAMI (Washington, Wyoming, Alaska, Montana, and Idaho) region Practice and Research Network. We will use two modalities for patient recruitment: (1) electronic health record (EHR) data extraction and (2) clinician referral. For EHR extraction, participating clinics will provide electronic health record (EHR) data that identify patients who meet the inclusion/exclusion criteria (all identifiable from the EHR except for the loneliness score). If patients have not opted out for research, they will be contacted by phone by the project manager (up to 3 contact attempts per eligible patient). For clinician referral, clinicians at participating clinics will be informed about the study via email and/or during a staff meeting and will have the opportunity to refer any patient who is on long-term opioid therapy to the study. For each patient we reach, we will inform them about the study and, if they are interested in participating, administer the 3-item UCLA loneliness scale over the phone to see if they are eligible (score of 6 or higher on the scale).

### Randomization

Prior to randomization, patients will be administered a baseline survey that includes a psychosis and cognitive screener, and demographic questions. After verification of eligibility, patients will be randomized using computer-generated random numbers to one of three groups: (1) CBT, (2) social prescribing or (3) usual care. There will be no stratification. The randomization will be blinded to the members of the study team responsible for data collection.

### Study intervention platform

Each active intervention (CBT and social prescribing) will consist of eight one hour, weekly group based sessions delivered via telehealth. Each group will consist of 6–10 participants using a HIPAA-compliant zoom platform allowing group members to see and hear each other.

### CBT intervention

The aim of the CBT intervention is to develop skills and strategies to change unhelpful social cognitions and behaviors, increase overall participation in social contexts, and improve perceived quality in existing relationships. We will adapt our CBT intervention from Ehde’s chronic pain CBT protocol and Käll’s loneliness CBT protocol, given no established CBT protocol that addresses both long-term opioid use and loneliness in primary care patients exists [38, 39]. The CBT intervention will include education about the connections between loneliness and substance use, practice of behavioral skills for effectively managing chronic pain and activating social participation, teaching of how to identify, challenge and modify unhealth thoughts and beliefs about pain along with their replacement with helpful thoughts, and ways to address anxiety with social interactions. The weekly group sessions will be delivered by a study interventionist who is a behavioral health clinician, mimicking a behavioral health referral from clinic. Each session will include at least one in-group skill building exercise. After each session, participants will be provided with a session summary and suggested home practice worksheet to facilitate practice of skills between sessions.

### Social prescribing intervention

The aim of the social prescribing intervention is to reduce loneliness by increasing opportunities for social access and connection. This intervention is modeled from the United Kingdom National Health Services social prescribing services. The primary support for this will be a social navigator, who does not need to be a behavioral health trained personnel and can be a peer or community member. During the initial group, the navigator will work with participants to complete an inventory of the participants’ existing social network, former social network, and interests. Using this, in the second group, the navigator will work with the participants to develop an action plan to promote, establish or re-establish social connections. The action plan will be referenced and refined throughout the course of the 8 weeks. The navigator will facilitate focused discussions on barriers that may arise, such as social anxiety, and strategies to overcome them. In the final week, the navigator will collaborate with participants to create a sustainability plan.

### Usual care

Participants assigned to the usual care arm will be notified of their group assignment and told that they can continue receiving care or seek care as they usually would. They will also be given a one-page handout about loneliness and local resources. There is low risk of contamination since those in usual care will not be aware of patients in the other arms of the study.

### Effectiveness outcomes

The effectiveness outcomes will be assessed at baseline, at completion of the intervention (at 8 weeks) and at 3 months following the completion of the intervention.

#### Loneliness

Our primary outcome is loneliness, as measured by the UCLA loneliness scale, Version 3 [40]. This scale consists of 20 items that ask patients to identify “how often they feel” followed by a positive or negative description of social interactions and perceptions, with participants ranking the frequency as never, rarely, sometimes and always. The scale is scored from 20 to 80 with 80 being the most lonely, and 20 being the least.

#### Opioid misuse

Opioid misuse is our main secondary outcome, as described in our conceptual framework, and will be measured using the Current Opioid Misuse Measure (COMM) [41, 42]. The COMM is a self-administered questionnaire that assesses past 30 day behaviors concerning for addiction or taking a medication in a way other than how it is prescribed using 16 items each scored on a 5-point Likert scale with total scores ranging from 0 to 64. A COMM score of 13 or higher is indicative of problematic drug use and risk for opioid use disorder [43].

#### Secondary outcomes

Our other secondary outcomes include function [44], substance use [45, 46], social connection [47], depression [48], anxiety [49, 50], and prescribed opioid use. The measures for each outcome can be found in Table 1.

**Table 1:** Effectiveness outcomes collected at baseline, post-intervention and 3 months post-intervention for randomized controlled trial

Outcome	Measure	Method of Collection
Loneliness	UCLA Loneliness Scale, Version 3	Patient report
Opioid Misuse	Current Opioid Misuse Measure	Patient report
Social Connection	Lubben Social Network Scale	Patient report
Substance Use	TAPS-1 (Tobacco, Alcohol, Prescription medication and other Substance use)	Patient report
Depression	PHQ-9	Patient report
Anxiety	GAD-7	Patient report
Physical Function	PROMIS physical functioning short form 6b	Patient report
Prescribed Opioid Use	Opioid type, dose and frequency (to calculate morphine equivalent dose)	EHR extraction

### Implementation outcomes

We propose using the RE-AIM (Reach, Effectiveness, Adoption, Implementation and Maintenance) framework to assess the feasibility of implementing a 3-arm trial [51, 52].

*Reach* – We will measure the proportion of patients who are reached and agree to participate in our study by tracking total patients reached and total patients enrolled. Specifically, we will track the demographic characteristics of gender, race/ethnicity, rurality and initial opioid dose to determine if specific subpopulations are more easily reached and enrolled in our study.

*Effectiveness* – see above for effectiveness outcomes.

*Adoption* – We will track the proportion of eligible clinics that agree to participate in the study and the number of eligible clinics that may be interested but decline to participate at this time. We will compare the clinic characteristics (size, rurality, patient demographics) of clinics that agreed to participate with those clinics that were eligible but declined participation. Furthermore, all study personnel who interact with participating clinics will keep notes on any interactions with clinic staff to measure level of engagement, which will be stratified by staff type, clinic type and patient population served.

*Implementation* – We will measure fidelity with both the CBT and social prescribing interventions. For the CBT intervention, the treatment fidelity protocol will consist of the level of adherence to the study manual with documentation from interventionists, fidelity checklist, and ongoing supervision meetings with the supervising investigators. We will also note any necessary adaptations that are made based on patients’ needs or expressed desires during the group sessions. Patients’ attendance and level of engagement in group sessions will also be documented by study interventionists. For the social prescribing intervention, we will track development of the patient narratives, network inventories and action plans, adherence to regular visits/check-ins with the social navigator, substantial variations in between patients in the intervention (i.e., visit durations, types of plans, etc.) and necessary adaptations based on patients’ needs or expressed desires.

*Maintenance* – We will measure if any change in outcomes at post-intervention is sustained at 3 months post-intervention for this trial. In a future pragmatic trial, we plan to measure sustainability at the practice/health system level.

### Qualitative interviews

Qualitative interviews will elucidate multiple elements from within the RE-AIM framework in greater detail. We will conduct 20 semi-structured interviews with patients (10 from the CBT intervention and 10 from the social prescribing intervention) at the conclusion of the intervention. We will recruit patients via phone with a $100 incentive offered to those who agree to and complete the interview (this incentive is in addition to other incentives offered to patients for completion of data measures). We will intentionally sample patients of diverse gender, race/ethnicity and geographic location, and if feasible, both patients who have attended the majority/all of the intervention sessions and those who have only attended a few (if any) of the sessions of the assigned intervention. We will ask patients about their experience of loneliness and how it has affected their chronic pain and opioid use, their experience of the interventions including any changes in their chronic pain, opioid use, and social connections that have occurred over the course of the treatment, history of opioid misuse and/or opioid use disorder (if any) and how this may have changed over the course of the interventions, thoughts about facilitators and barriers to accessing the intervention and suggestions for intervention improvements.

### Data collection

We will collect the outcome measures from patients in the week post intervention and 3 months post intervention (or for those in the usual care arm 10 weeks after initial data collection and then 3 months following that). Data collection will take place via phone, email or mail depending on the patient preference indicated in their baseline data collection. Patients will be given a $50 gift card incentive for each data collection time point.

### Power analysis

We plan to recruit 102 patients on long-term opioid therapy based on a sample size calculation allowing us to detect 6-point changes or more in the UCLA loneliness scale (Version 3). The 6-point change was determined by the mean loneliness score change from a previously published meta-analyses of psychological interventions for loneliness [31]. For the sample size calculation, we also assumed a significance level of 0.05, a desired power of 0.80, and a standard deviation of 8.0 (from weighted average from the original validation of the UCLA loneliness scale). Based on this, we will require 28 patients per arm. Assuming an 80% retention rate, we plan to recruit 34 patients per arm (or 102 patients total).

### Quantitative analysis

The analysis population will include all randomized patients. Patients will be analyzed according to their assigned intervention group, regardless of what treatment they receive (intent to treat analysis). Descriptive statistics (mean and standard deviation) will be used to summarize the distribution of the primary outcome (loneliness), and secondary outcomes (opioid misuse, depression, anxiety, functionality and opioid dose) at baseline, immediately following the intervention and 3 months following the intervention. Linear mixed models will be used to test the association between the intervention arm and the change in the primary outcome and secondary outcomes across the study period. Since we expect that the effect from the intervention takes places immediately after implementation, we will include the intervention arm (control arm as the reference), an indicator for the post-treatment time points (immediately following the intervention and 3 months after) as well as their interaction as independent variables. Intervention assignment will be modeled as a fixed effect, and individual participants will be modeled as a random effect. Each model will contain a random intercept to account for the subject-specific correlation in outcome. In a secondary analysis, we will add a contrast variable to test the maintenance of outcomes between the second and third time points.

Subgroup analyses, which will be exploratory in nature, will be performed using the same modeling technique to assess the heterogeneity of treatment effects across subgroups of patients defined by age (grouped by tertiles), gender (male, female and nonbinary), rurality (rural urban continuum codes), initial opioid MME (calculated using the Centers for Disease Control and Prevention’s data file on opioid analgesic morphine milligram equivalents and grouped by tertiles) and initial loneliness score (grouped by tertiles).

Missing data mechanisms will be examined by correlating the proportion of missing data with key baseline characteristics. Multiple imputation by chained equations will be applied to account for loss-to-follow-up. Inferences will be drawn based on two-sided *p*-value of 0.05. Given that all participants are randomly assigned to one of the three intervention arms, we do not expect substantial bias in unadjusted analysis results. Hence, we do not plan to include control variables in our statistical modelling.

### Qualitative analysis

For the qualitative interviews, an immersion-crystallization process will be used to identify key themes in the data [53]. The themes will describe patient experiences of the interventions, effects of the interventions on their loneliness, chronic pain and opioid use, and patients’ perceived facilitators and barriers to accessing the intervention. A codebook will be created that combines emergent and a priori themes derived from the interview guide. Two coders will independently code each transcript. Coders will meet weekly throughout the coding process. If any new themes emerge during the coding process, these will be discussed as a team and may be added to the codebook. Any disagreement in coding will be resolved by consensus including a third coder. Once all interview transcripts have been coded, the coders will meet to discuss the themes, search for patterns and overarching interpretations in the themes, seek alternative interpretations, and ask whether the themes and patterns may be interpreted in a different manner. This process will continue until no further interpretations are generated.

### Ethics and data monitoring

Since this trial is low risk, no data safety monitoring board was required. Adverse events will be monitored by the Principal Investigator and reported to the funder. There is no plan for auditing trial conduct.

The study has been approved by the University of Washington Institutional Review Board, which will serve as the single IRB of record. The principal investigator will be responsible for communicating all important protocol modifications to relevant parties. Research coordinators will be responsible for obtaining informed consent. Personal information will be stored in a secure, password protected database and study personnel will have access to the full deidentified data set. Trial results will be disseminated to all study participants in a user-friendly one page brief at the end of the study.

### Dissemination and future steps

We plan to disseminate our findings in a peer-reviewed journal and also at primary care, addiction and behavioral health national conferences. We will also develop a 1-pager to disseminate the results of the study back to participants, participating clinics and to other interested clinics within the WPRN.

## Discussion

Our study targets loneliness and social connection with the goal of reducing opioid misuse, risk of harm for long-term opioid therapy and incidence of substance use disorder. It is particularly relevant with the growing prevalence of loneliness with a parallel rise in drug overdoses in the United States [17, 54]. If successful, our study can help build meaningful social connections and foster community for individuals who are at high risk of harm from opioids and other substances.

Limitations to our study include potential selection bias, since those who choose to engage in our study could be more motivated than the general population. Furthermore, we recognize that our interventionists who are study personnel may not directly mimic clinic personnel although our patients will be recruited from primary care practices and the process of getting to the study interventionist will mimic a typical referral process. Finally, our dependent secondary outcome, COMM, measures for risk for opioid misuse and does not measure actual behavior of misuse or downstream outcomes directly.

Our study tests the effectiveness of two evidence-based interventions for loneliness for patients with long-term opioid use from primary care practices. If successful, it could serve as the foundation for a sustainable primary care interventions to ameliorate loneliness to reduce opioid misuse and opioid use disorder.

## Data Availability

No datasets were generated or analysed during the current study.

## References

[1] Ingram I, Kelly PJ, Deane FP, Baker AL, Goh MC, Raftery DK, Dingle GA. Loneliness among people with substance use problems: a narrative systematic review. Drug Alcohol Rev. 2020;39(5):447–83. 32314504 10.1111/dar.13064

[2] National Institute on Drug Abuse. Enhancing Social Connectedness and Ameliorating Loneliness to Prevent and Treat SUD and Support Recovery 2022 [cited 2022 13 Jul]. https://grants.nih.gov/grants/guide/rfa-files/RFA-DA-23-010.html

[3] Pai N, Vella S-L. COVID-19 and loneliness: a rapid systematic review. Australian New Z J Psychiatry. 2021;55(12):1144–56. 34256632 10.1177/00048674211031489

[4] Killgore WD, Cloonan SA, Taylor EC, Dailey NS. Loneliness: a signature mental health concern in the era of COVID-19. Psychiatry Res. 2020;290:113117. 32480121 10.1016/j.psychres.2020.113117 PMC7255345

[5] Loades ME, Chatburn E, Higson-Sweeney N, Reynolds S, Shafran R, Brigden A, Linney C, McManus MN, Borwick C, Crawley E. Rapid systematic review: the impact of social isolation and loneliness on the mental health of children and adolescents in the context of COVID-19. J Am Acad Child Adolesc Psychiatry. 2020;59(11):1218–39. e3. 32504808 10.1016/j.jaac.2020.05.009 PMC7267797

[6] Ernst M, Niederer D, Werner AM, Czaja SJ, Mikton C, Ong AD, Rosen T, Brähler E, Beutel ME. Loneliness before and during the COVID-19 pandemic: a systematic review with meta-analysis. Am Psychol. 2022. 10.1037/amp0001005. No Pagination Specified-No Pagination Specified. 35533109 10.1037/amp0001005 PMC9768682

[7] Kelley MA, Lucas J, Stewart E, Goldman D, Doctor JN. Opioid-related deaths before and after COVID-19 stay-at-home orders in Los Angeles County. Drug Alcohol Depend. 2021;228:109028. 10.1016/j.drugalcdep.2021.109028. Epub 20210902. 34500239 10.1016/j.drugalcdep.2021.109028 PMC8411574

[8] Kuehn BM. Accelerated overdose deaths linked with COVID-19. JAMA. 2021;325(6):523. 10.1001/jama.2021.0074 33560330 10.1001/jama.2021.0074

[9] Linas BP, Savinkina A, Barbosa C, Mueller PP, Cerdá M, Keyes K, Chhatwal J. A clash of epidemics: impact of the COVID-19 pandemic response on opioid overdose. J Subst Abuse Treat. 2021;120:108158. 10.1016/j.jsat.2020.108158. Epub 2020/10/06. 33298298 10.1016/j.jsat.2020.108158 PMC7536128

[10] National Center for Health Statistics. Provisional Drug Overdose Death Counts 2022 [cited 2022 Apr 24]. https://www.cdc.gov/nchs/nvss/vsrr/drug-overdose-data.htm

[11] National Institute on Drug Abuse. Overdose Death Rates 2022 [cited 2022 Apr 24]. https://nida.nih.gov/drug-topics/trends-statistics/overdose-death-rates

[12] Dowell D, Haegerich TM, Chou R. CDC guideline for prescribing opioids for chronic pain—United States, 2016. JAMA. 2016;315(15):1624–45. 26977696 10.1001/jama.2016.1464 PMC6390846

[13] Lee B, Yang K-C, Kaminski P, Peng S, Odabas M, Gupta S, Green HD, Jr, Ahn Y-Y, Perry BL. Substitution of nonpharmacologic therapy with opioid prescribing for Pain during the COVID-19 pandemic. JAMA Netw Open. 2021;4(12):e2138453–e. 10.1001/jamanetworkopen.2021.38453 34889946 10.1001/jamanetworkopen.2021.38453 PMC8665369

[14] Currie JM, Schnell MK, Schwandt H, Zhang J. Prescribing of opioid analgesics and Buprenorphine for Opioid Use Disorder during the COVID-19 pandemic. JAMA Netw Open. 2021;4(4):e216147–e. 10.1001/jamanetworkopen.2021.6147 33856474 10.1001/jamanetworkopen.2021.6147 PMC8050741

[15] Manchikanti L, Vanaparthy R, Atluri S, Sachdeva H, Kaye AD, Hirsch JA. COVID-19 and the opioid epidemic: two Public Health emergencies that Intersect with Chronic Pain. Pain Therapy. 2021;10(1):269–86. 10.1007/s40122-021-00243-2 33718982 10.1007/s40122-021-00243-2 PMC7955940

[16] Silva MJ, Kelly Z. The escalation of the opioid epidemic due to COVID-19 and resulting lessons about treatment alternatives. Am J Manag Care. 2020;26(7):e202–4. 32672917 10.37765/ajmc.2020.43386

[17] Murthy V. Our epidemic of loneliness and isolation: the U.S. Surgeon General’s Advisory on the Healing effects of social connection and community. Office of the U.S. Surgeon General; 2023. 37792968

[18] Gerst-Emerson K, Jayawardhana J. Loneliness as a public health issue: the impact of loneliness on health care utilization among older adults. Am J Public Health. 2015;105(5):1013–9. 10.2105/ajph.2014.302427. Epub 20150319. 25790413 10.2105/ajph.2014.302427 PMC4386514

[19] Taube E, Kristensson J, Sandberg M, Midlöv P, Jakobsson U. Loneliness and health care consumption among older people. Scand J Caring Sci. 2015;29(3):435–43. 10.1111/scs.12147. Epub 20140514. 24826811 10.1111/scs.12147

[20] Geller J, Janson P, McGovern E, Valdini A. Loneliness as a predictor of hospital emergency department use. J Fam Pract. 1999;48(10):801–4. PubMed PMID: 12224678. 12224678

[21] Molloy GJ, McGee HM, O’Neill D, Conroy RM. Loneliness and emergency and planned hospitalizations in a community sample of older adults. J Am Geriatr Soc. 2010;58(8):1538-41. Epub 20100714. 10.1111/j.1532-5415.2010.02960.x. PubMed PMID: 20646104. 10.1111/j.1532-5415.2010.02960.x 20646104

[22] Green LA, Fryer GE Jr., Yawn BP, Lanier D, Dovey SM. The ecology of medical care revisited. N Engl J Med. 2001;344(26):2021–5. 10.1056/nejm200106283442611. PubMed PMID: 11430334. 11430334 10.1056/nejm200106283442611

[23] Levy B, Paulozzi L, Mack KA, Jones CM. Trends in opioid analgesic–prescribing rates by specialty, US, 2007–2012. Am J Prev Med. 2015;49(3):409–13. 25896191 10.1016/j.amepre.2015.02.020 PMC6034509

[24] Mills S, Torrance N, Smith BH. Identification and Management of Chronic Pain in Primary Care: a review. Curr Psychiatry Rep. 2016;18(2):22. 10.1007/s11920-015-0659-9. PubMed PMID: 26820898; PMCID: PMC4731442. 26820898 10.1007/s11920-015-0659-9 PMC4731442

[25] Mullen RA, Tong S, Sabo RT, Liaw WR, Marshall J, Nease DE Jr., Krist AH, Frey JJ. 3rd. Loneliness in primary care patients: a prevalence study. Ann Fam Med. 2019;17(2):108–15. 10.1370/afm.2358. PubMed PMID: 30858253; PMCID: PMC6411405. 30858253 10.1370/afm.2358 PMC6411405

[26] Tong S, Mullen RA, Hochheimer CJ, Sabo RT, Liaw WR, Nease DE Jr., Krist AH, Frey JJ 3. Geographic characteristics of loneliness in primary care. Ann Fam Med. 2019;17(2):158–60. 10.1370/afm.2364. PubMed PMID: 30858259; PMCID: PMC6411395. 10.1370/afm.2364 PMC6411395 30858259

[27] Sirois FM, Owens J. A meta-analysis of loneliness and use of primary health care. Health Psychol Rev. 2021;1–18. 10.1080/17437199.2021.1986417 10.1080/17437199.2021.1986417 34581240

[28] American Academy of Family Physicians. Family Physician, Definition 2019 [cited 2022 Apr 6]. https://www.aafp.org/about/policies/all/family-physician-definition.html#Family Physician, Definition

[29] McLellan AT, Koob GF, Volkow ND. Preaddiction—A Missing Concept for Treating Substance Use disorders. JAMA Psychiatry. 2022. 10.1001/jamapsychiatry.2022.1652 35793096 10.1001/jamapsychiatry.2022.1652

[30] Volkow N. Time to Start Talking About Pre-Addiction 2022 [cited 2022 29 Jul]. https://nida.nih.gov/about-nida/noras-blog/2022/07/time-to-start-talking-about-pre-addiction

[31] Masi CM, Chen H-Y, Hawkley LC, Cacioppo JT. A meta-analysis of interventions to reduce loneliness. Pers Soc Psychol Rev. 2011;15(3):219–66. Epub 2010/08/17. doi: 10.1177/1088868310377394. PubMed PMID: 20716644. 20716644 10.1177/1088868310377394 PMC3865701

[32] Hickin N, Käll A, Shafran R, Sutcliffe S, Manzotti G, Langan D. The effectiveness of psychological interventions for loneliness: a systematic review and meta-analysis. Clin Psychol Rev. 2021;88:102066. 34339939 10.1016/j.cpr.2021.102066

[33] National Academies of Sciences EaM. Social isolation and loneliness in older adults: opportunities for the Health Care System. Washington, DC: National Academies; 2020. 32510896

[34] Emerson K, Boggero I, Ostir G, Jayawardhana J. Pain as a risk factor for loneliness among older adults. J Aging Health. 2018;30(9):1450–61. 28728466 10.1177/0898264317721348

[35] Jaremka LM, Andridge RR, Fagundes CP, Alfano CM, Povoski SP, Lipari AM, Agnese DM, Arnold MW, Farrar WB, Yee LD. Pain, depression, and fatigue: loneliness as a longitudinal risk factor. Health Psychol. 2014;33(9):948. 23957903 10.1037/a0034012 PMC3992976

[36] Karos K, McParland JL, Bunzli S, Devan H, Hirsh A, Kapos FP, Keogh E, Moore D, Tracy LM, Ashton-James CE. The social threats of COVID-19 for people with chronic pain. Pain. 2020;161(10):2229–35. 10.1097/j.pain.0000000000002004. PubMed PMID: 32694381. 32694381 10.1097/j.pain.0000000000002004 PMC7382418

[37] Loeffler A, Steptoe A. Bidirectional longitudinal associations between loneliness and pain, and the role of inflammation. Pain. 2021;162(3):930. 32960533 10.1097/j.pain.0000000000002082 PMC7886943

[38] Ehde DM, Alschuler KN, Day MA, Ciol MA, Kaylor ML, Altman JK, Jensen MP. Mindfulness-based cognitive therapy and cognitive behavioral therapy for chronic pain in multiple sclerosis: a randomized controlled trial protocol. Trials. 2019;20(1):774. 10.1186/s13063-019-3761-1. Epub 20191227. 31882017 10.1186/s13063-019-3761-1 PMC6935157

[39] Käll A, Bäck M, Welin C, Åman H, Bjerkander R, Wänman M, Lindegaard T, Berg M, Moche H, Shafran R, Andersson G. Therapist-guided internet-based treatments for loneliness: a Randomized Controlled three-arm trial comparing cognitive behavioral therapy and interpersonal psychotherapy. Psychother Psychosom. 2021;90(5):351–8. Epub 20210628. doi: 10.1159/000516989. PubMed PMID: 34182552. 34182552 10.1159/000516989

[40] Russell DW. UCLA Loneliness Scale (Version 3): reliability, validity, and factor structure. J Pers Assess. 1996;66(1):20–40. 8576833 10.1207/s15327752jpa6601_2

[41] Butler SF, Budman SH, Fanciullo GJ, Jamison RN. Cross validation of the current opioid misuse measure to monitor chronic pain patients on opioid therapy. Clin J Pain. 2010;26(9):770-6. 10.1097/AJP.0b013e3181f195ba. PubMed PMID: 20842012. 10.1097/AJP.0b013e3181f195ba PMC2955853 20842012

[42] Butler SF, Budman SH, Fernandez KC, Houle B, Benoit C, Katz N, Jamison RN. Development and validation of the Current Opioid Misuse Measure. Pain. 2007;130(1–2):144 – 56. Epub 2007/05/09. 10.1016/j.pain.2007.01.014. PubMed PMID: 17493754. 10.1016/j.pain.2007.01.014 PMC1950245 17493754

[43] Meltzer EC, Rybin D, Saitz R, Samet JH, Schwartz SL, Butler SF, Liebschutz JM. Identifying prescription opioid use disorder in primary care: diagnostic characteristics of the current opioid misuse measure (COMM). Pain. 2011;152(2):397–402. PubMed PMID: 21177035; PMCID: PMC3027065. 21177035 10.1016/j.pain.2010.11.006 PMC3027065

[44] Amtmann D, Cook KF, Jensen MP, Chen WH, Choi S, Revicki D, Cella D, Rothrock N, Keefe F, Callahan L, Lai JS. Development of a PROMIS item bank to measure pain interference. Pain. 2010;150(1):173–82. PubMed PMID: 20554116; PMCID: PMC2916053. 20554116 10.1016/j.pain.2010.04.025 PMC2916053

[45] Gryczynski J, McNeely J, Wu LT, Subramaniam GA, Svikis DS, Cathers LA, Sharma G, King J, Jelstrom E, Nordeck CD, Sharma A, Mitchell SG, O’Grady KE, Schwartz RP. Validation of the TAPS-1: a four-item Screening Tool to identify unhealthy substance use in primary care. J Gen Intern Med. 2017;32(9):990–6. 10.1007/s11606-017-4079-x. Epub 20170526. 28550609 10.1007/s11606-017-4079-x PMC5570743

[46] McNeely J, Wu LT, Subramaniam G, Sharma G, Cathers LA, Svikis D, Sleiter L, Russell L, Nordeck C, Sharma A, O’Grady KE, Bouk LB, Cushing C, King J, Wahle A, Schwartz RP. Performance of the Tobacco, Alcohol, prescription medication, and other Substance Use (TAPS) Tool for Substance Use Screening in Primary Care patients. Ann Intern Med. 2016;165(10):690–9. 10.7326/m16-0317. Epub 20160906. 27595276 10.7326/m16-0317 PMC5291717

[47] Buckley TD, Becker TD, Burnette D. Validation of the abbreviated Lubben Social Network Scale (LSNS-6) and its association with self‐rated health amongst older adults in Puerto Rico. Health Soc Care Commun. 2022;30(6):e5527–38. 10.1111/hsc.13977 36039906

[48] Arroll B, Goodyear-Smith F, Crengle S, Gunn J, Kerse N, Fishman T, Falloon K, Hatcher S. Validation of PHQ-2 and PHQ-9 to screen for major depression in the primary care population. Annals Family Med. 2010;8(4):348–53. 10.1370/afm.1139 PMC2906530 20644190

[49] Löwe B, Decker O, Müller S, Brähler E, Schellberg D, Herzog W, Herzberg PY. Validation and standardization of the generalized anxiety disorder screener (GAD-7) in the general population. Med Care. 2008:266–74. 10.1097/MLR.0b013e318160d093 18388841

[50] Spitzer RL, Kroenke K, Williams JB, Löwe B. A brief measure for assessing generalized anxiety disorder: the GAD-7. Arch Intern Med. 2006;166(10):1092–7. 16717171 10.1001/archinte.166.10.1092

[51] Glasgow RE, Harden SM, Gaglio B, Rabin B, Smith ML, Porter GC, Ory MG, Estabrooks PA. RE-AIM planning and evaluation Framework: adapting to New Science and Practice with a 20-Year review. Front Public Health. 2019;7. 10.3389/fpubh.2019.00064 10.3389/fpubh.2019.00064 PMC6450067 30984733

[52] Holtrop JS, Estabrooks PA, Gaglio B, Harden SM, Kessler RS, King DK, Kwan BM, Ory MG, Rabin BA, Shelton RC. Understanding and applying the RE-AIM framework: clarifications and resources. J Clin Translational Sci. 2021;5(1). 10.1017/cts.2021.789 PMC8327549 34367671

[53] Ryan GW, Bernard HR. Techniques to identify themes. Field Methods. 2003;15(1):85–109. 10.1177/1525822x02239569 10.1177/1525822x02239569

[54] Spencer M, Minino A, Warner M. Drug Overdose Deaths in the United States, 2001–2021. Atlanta, GA: Centers for Disease Control and Prevention; 2022. NCHS Data Brief No. 457. 36598401

